# Alternative polyadenylation and dynamic 3′ UTR length is associated with polysome recruitment throughout the cardiomyogenic differentiation of hESCs

**DOI:** 10.3389/fmolb.2024.1336336

**Published:** 2024-02-06

**Authors:** Aruana F. F. Hansel-Frose, Jens Allmer, Marcel Friedrichs, Hellen Geremias dos Santos, Bruno Dallagiovanna, Lucía Spangenberg

**Affiliations:** ^1^ Laboratory of Basic Stem Cell Biology, Carlos Chagas Institute, Oswaldo Cruz Foundation (FIOCRUZ/PR), Curitiba, Brazil; ^2^ Department of Medical Informatics and Bioinformatics, University of Applied Sciences Ruhr West, Mülheim, Germany; ^3^ Bioinformatics and Medical Informatics Department, University of Bielefeld, Bielefeld, Germany; ^4^ Carlos Chagas Institute, Oswaldo Cruz Foundation (FIOCRUZ/PR), Curitiba, Brazil; ^5^ Bioinformatics Unit, Pasteur Institute of Montevideo, Montevideo, Uruguay; ^6^ Departamento Basico de Medicina, Hospital de Clinicas, Universidad de la República (Udelar), Montevideo, Uruguay

**Keywords:** human embryonic stem cells, miRNA, polysome profiling, gene regulatory network, cardiomyocytes, cardiomyogenesis

## Abstract

Alternative polyadenylation (APA) increases transcript diversity through the generation of isoforms with varying 3′ untranslated region (3′ UTR) lengths. As the 3′ UTR harbors regulatory element target sites, such as miRNAs or RNA-binding proteins, changes in this region can impact post-transcriptional regulation and translation. Moreover, the APA landscape can change based on the cell type, cell state, or condition. Given that APA events can impact protein expression, investigating translational control is crucial for comprehending the overall cellular regulation process. Revisiting data from polysome profiling followed by RNA sequencing, we investigated the cardiomyogenic differentiation of pluripotent stem cells by identifying the transcripts that show dynamic 3′ UTR lengthening or shortening, which are being actively recruited to ribosome complexes. Our findings indicate that dynamic 3′ UTR lengthening is not exclusively associated with differential expression during cardiomyogenesis but rather with recruitment to polysomes. We confirm that the differentiated state of cardiomyocytes shows a preference for shorter 3′ UTR in comparison to the pluripotent stage although preferences vary during the days of the differentiation process. The most distinct regulatory changes are seen in day 4 of differentiation, which is the mesoderm commitment time point of cardiomyogenesis. After identifying the miRNAs that would target specifically the alternative 3′ UTR region of the isoforms, we constructed a gene regulatory network for the cardiomyogenesis process, in which genes related to the cell cycle were identified. Altogether, our work sheds light on the regulation and dynamic 3′ UTR changes of polysome-recruited transcripts that take place during the cardiomyogenic differentiation of pluripotent stem cells.

## 1 Introduction

There are a few ways by which the repertoire of transcripts in a cell can be increased from the expression of the same set of genes. Alternative polyadenylation (APA) is a process that plays an important role in transcript diversity during post-transcriptional regulation. With alternative polyadenylation sites (PASs), isoforms of the same gene are generated, which differ by the length of the untranslated region at the 3′ end (3′ UTR). Subsequently, the 3′ UTR of an mRNA contributes to the post-transcriptional regulation of this transcript since it can impact protein localization and/or abundance. In addition, the region can contain several regulatory elements, such as microRNA target sites. Once the length of this region is changed through APA, it can, therefore, influence or modify the said regulatory process in the cell (Turner et al., 2018; Mayr, 2019; Sommerkamp et al., 2021).

Alternative polyadenylated transcripts can be differentially expressed between various conditions, not only during diseases but also in healthy tissues and throughout development (Gruber and Zavolan, 2019; MacDonald, 2019). Studies have shown that differential APA expression can be seen during stem cell differentiation (Spangenberg et al., 2013; Sommerkamp et al., 2021). The dynamic APA site usage can change depending on the cell state: active proliferating stem or progenitor cells exhibit short 3′ UTRs, while long 3′ UTRs are mostly seen in quiescent stem cells (Sommerkamp et al., 2021).

Since APA events can influence protein expression, the study of translational control is important to understand the whole regulation process that takes place inside the cell. Translational control can be regulated by the association or dissociation of transcripts to polysome complexes, which are composed of various ribosomes bound to the same transcript being actively translated. Using the polysome profiling technique followed by high-throughput transcriptome sequencing, it is possible to specifically identify which transcripts are bound or unbound to polysomes, allowing a better understanding of the translational control of the cell (Schwanhüusser et al., 2011; Tebaldi et al., 2012). In the context of stem cell research, this strategy has the potential to enhance our understanding of the dynamics of post-transcriptional regulation during cell fate commitment (Pereira et al., 2018; 2019).

Considering that stem cells’ self-renewal and differentiation potential are interesting characteristics regarding regenerative medicine (Trounson and McDonald, 2015), understanding the process of differentiation into various tissues is essential for addressing diseases in the future. According to the World Health Organization, cardiovascular disease is still the global leading cause of death, which supports the need for more research in cardiomyogenic differentiation to improve possible therapies (Bhagavati, 2015; Trounson and McDonald, 2015). Pluripotent stem cells are good models for cardiomyogenesis research, and they can differentiate into cardiomyocytes while reflecting the development of the embryonic heart (Beqqali et al., 2006; Brade et al., 2013; Leitolis et al., 2019). Notably, pluripotent stem cells are also good candidates for future applications in cell therapy (Trounson and McDonald, 2015; Zhang et al., 2015).

As previously shown by our group, during cardiomyogenic differentiation, there is extensive post-transcriptional regulation, fine-tuned by not only coding but also non-coding transcripts bound to polysomes (Pereira et al., 2019; 2020). To add a new layer of understanding to this process, in this work, we investigated the same process while focusing on the dynamic 3′ UTR length changes due to APA through the days of differentiation.

Here, we aim to assess whether post-transcriptional regulation of mRNA is influenced by dynamic UTR lengthening and shortening through the events of alternative polyadenylation. This event might alter the availability of miRNA target sites in 3′ UTR during human embryonic stem cell (hESC) cardiac differentiation.

## 2 Materials and methods

### 2.1 RNA sequencing analysis

The hESC line hES-NKX2-5^eGFP/w^ differentiation to cardiomyocytes, RNA sequencing, and polysome profiling were previously carried out by our group (Pereira et al., 2018). In summary, the transcriptomes of both fractions that were free of polysomes and polysome-bound from the days of cardiomyogenic differentiation of days 0, 1, 4, 9, and 15 were sequenced. The reanalysis of the unstranded RNA-seq began with quality control with FastQC (Andrews, 2015) and trimming of reads with Trim Galore (v. 0.4.0) (Krueger, 2015). The alignment of reads was performed with HISAT2 (v. 2.1.0) (Kim et al., 2015) against the human genome hg38/GRCh38 v.103, with read counts performed by HTSeq (v. 0.11.1) (Anders et al., 2015). Differential gene expression was calculated with DESeq2 (v. 1.24.0) (Love et al., 2014) at a gene level considering an adjusted *p*-value cutoff of 0.05 and log2FoldChange cutoff of |2|. The counts per million (CPM) normalization of reads was carried out in R (R Team, 2018).

### 2.2 Differential 3′ UTR length identification

Both the identification of the 3′ UTR sequence, with the start and end sites of alternative polyadenylation, and the differential 3′ UTR lengthening were performed with APAtrap (Ye et al., 2018). First, the alignment files and the human genome hg38/GRCh38 v.103 were converted to bedGraph using bedtools (v. 2.28.0) (Quinlan and Hall, 2010). The identification of annotated 3′ UTRs and the prediction of 3′ UTR lengthening and shortening through the identification of alternative polyadenylation sites were performed based on the annotation of the human genome hg38/GRCh38 v.103. The default 3′ UTR annotation provided by Ensembl was used, and APAtrap calculated the lengthening and shortening of the 3′ UTR at the isoform level.

The comparison of differential 3′ UTR length usage executed by APAtrap was calculated considering day 0 of differentiation as the control, such as D0P vs. D15P, D0P vs. D9P, D0P vs. D4P, and D0P vs. D1P. In addition, each sample’s replicates 1, 2, and 3 were considered as part of one group of the day of cardiomyogenic differentiation. The results from APAtrap indicated the expressed transcript ID, gene ID, additional predicted APA sites if available, percentage difference between the groups (perc_diff), and preference for longer or shorter 3′ UTR through the Pearson correlation coefficient (r).

The results were filtered according to APAtrap’s default requirements for genes that show differential APA site usage, which considers the threshold for the adjusted *p*-value of 0.05 and the percentage difference (perc_diff) of expression between the control and the group of more than 20% (Ye et al., 2018).

The results were subsequently filtered using the differential gene expression obtained previously from DESeq2 (Love et al., 2014). Since differential expression was carried out at a gene level with DESeq2 and the 3′ UTR analysis was conducted at a transcript level with APAtrap, we filtered the dynamic 3′ UTR transcripts when the gene was a protein-coding gene, and it was significantly upregulated or downregulated according to the DESeq2 differential expression analysis (at 0.05 significance level).

The genes with log2FoldChange ≥2 were considered upregulated genes, while the ones with log2FoldChange ≤ −2 were considered downregulated genes. Both groups were filtered for the adjusted *p*-value (padj) ≤ 0.05.

In order to identify the preference for the use of longer or shorter 3′ UTR, we observed the Pearson correlation coefficient (r) that also results from the APAtrap analysis. In detail, r < 0 indicates a preference for that transcript with short 3′ UTR in polysome-bound group 2 in relation to D0, and r > 0 indicates a preference for long 3′ UTR transcripts in polysome-bound group 2 in relation to D0.

### 2.3 Relation between differential gene expression and differential 3′ UTR length

The identification of the genes that simultaneously showed differential gene expression and differential 3′ UTR lengthening usage was carried out by plotting log2FoldChange and the Pearson correlation coefficient, discerning the 3′ UTR lengthening and shortening by r > 0 and r < 0, respectively. The comparison of the days of differentiation considered day 0 as the control. The genes were divided into four distinct groups: upregulated genes that also showed 3′ UTR lengthening, upregulated genes that also showed 3′ UTR shortening, downregulated genes that also showed 3′ UTR lengthening, and downregulated genes that also showed 3′ UTR shortening. Venn diagrams of these common transcripts or genes between the days of differentiation were created with InteractiVenn (Heberle et al., 2015).

### 2.4 Descriptive statistics of polysome recruitment

To calculate the recruitment of polysomes, raw reads were normalized to CPM and filtered for genes that have at least one CPM in at least three samples. The relative frequency (RF) of polysome recruitment was calculated as the polysomal fraction divided by the total RNA (polysomal + free of ribosomes) for each time point, R0, R1, R4, R9, and R15. Then, relative risk (RR) was calculated considering the RF of each time point divided by the RF of day zero (D0) as the control. Therefore, the interpretation of polysomal recruitment through the RF is in relation to the same day of differentiation, and through the RR, it is in relation to D0. When RR > 1, the gene shows a higher risk of being recruited into the polysomal fraction of the day of differentiation compared to D0. When RR < 1, the gene shows a lower risk of being recruited to polysomes compared to D0. When RR = 1, there is no difference in the risk of being recruited to polysomes in one day compared to D0. Finally, the results were filtered to transcripts that also showed dynamic 3′ UTR lengthening through the Pearson correlation coefficient (r) to associate the short 3′ UTR or long 3′ UTR changes to the recruitment of polysomes. Calculations were performed in R (R Team, 2018).

### 2.5 miRNA targets in 3′ UTR sequences

We searched for miRNA target sites in the 3′ UTR sequence of the differentially expressed genes that simultaneously showed differential expression and differential 3′ UTR length. To predict miRNA target sites in the 3′ UTR, the sequence from the 3′ UTR coordinates was retrieved from the human genome hg38/GRCh38 v.103, and the compilation of human miRNAs seed sites was retrieved from miRBase. The prediction between the two datasets was executed with psRNATarget (Dai et al., 2018). Afterward, the miRNAs–target matches were filtered, maintaining only the miRNAs expressed in each specific day of cardiomyogenic differentiation, using the miRNAome of cardiac differentiation as a reference (Garate et al., 2018). Relevant miRNAs were filtered according to a log2FoldChange of |2| and adjusted *p*-value of |0.05|. *In silico* confirmations of miRNA regulations were carried out in miRWalk and the Encyclopedia of RNA Interactome (ENCORI) (Li et al., 2014; Sticht et al., 2018).

### 2.6 Gene regulatory networks

Differentially expressed genes, their miRNA targets, and their corresponding APA isoforms were visualized in a gene regulatory network using igraph in R (Csardi and Nepusz, 2006; R Core Team, 2018). The details are given as follows.

First, the relevant data were merged in R (R Team, 2018) so that the following related columns were available in the same dataframe: the significant differentially expressed genes (log2FoldChange |2| and padj ≤0.05), their significantly expressed APA isoforms (perc_diff greater than 20% and *p*-value ≤0.05), and their respective significant miRNA targets (miRNAs that were differentially expressed with a log2FoldChange cutoff of |2| and padj ≤0.05, only when both miRNA and the gene target were expressed in the same day of cardiomyogenic differentiation). The dataframe was then adapted to a network format with the following specificities: the name vertex as the microRNAs, gene as the target gene, and UTR vertex as the APA isoform. A non-directed graph named “g” was created using igraph (Csardi and Nepusz, 2006) with the function graph_from_data_frame with the directed option as “False.” Labels of “miRNA,” “DNA” for the gene targets, and “mRNA” for the APA isoforms to the vertex were then added to the object V(g) of the graph. The log2FoldChange values were added to the corresponding vertex (miRNA, DNA, or mRNA) of the logFC label of the graph vertex (V(g)$logFC). Similarly, the short and long 3′ UTR attributes were added to the correct APA isoform through the r values obtained from APAtrap and incorporated into the graph object vertex with the label V(g)$r. Colors were attributed considering the values of the log2FoldChange, with dark blue for upregulated genes, light blue for downregulated genes, dark purple for upregulated miRNAs, and light purple for downregulated miRNAs. Edges between the vertex were colored considering short 3′ UTRs as salmon (when r < 0) and long 3′ UTR as blue when r > 0. The network was visualized in the R environment through an interactive interface using the function tkplot from igraph (Csardi and Nepusz, 2006) to plot the graph object “g.”

### 2.7 Visualization of alignments

The alignment of the alternative transcripts and their respective target miRNAs was visualized in the Integrative Genomics Viewer (IGV) version 2.9.4 (Thorvaldsdóttir et al., 2013). First, the coordinates of the miRNA target site on the 3′ UTRs, obtained from the previous analysis with psRNATarget (Dai et al., 2018), were converted to a BED file in the R environment (R Core Team, 2018). They were uploaded for visualization in IGV, together with the alignment files of the samples and the genome annotation from Ensembl version hg38.

## 3 Results

### 3.1 Cardiomyogenic differentiation of the hESC is analyzed through polysome profiling and transcriptome sequencing

Previously, the hESC line hES-NKX2-5^eGFP/w^ was differentiated into cardiomyocytes following a 5-time-point developmentally staged protocol that reaches the cardiac mesoderm stage at days 3 and 4 and the cardiomyocyte phenotype at day 15 (Pereira et al., 2018) (Supplementary Figure S1A). These days correspond to the pluripotency stage (D0), aggregation of embryoid bodies (D1), cardiac mesodermal stage (D4), cardiac progenitor (D9), and finally, cardiomyocytes (D15), respectively. We reanalyzed the RNA sequencing of the polysome profile previously published by our group (Pereira et al., 2018; Pereira et al., 2019), starting with the realignment of the sequenced transcriptomes in the recent version of the human genome GRCh38 v.103. (Supplementary Figure S1A, Supplementary Table S1). On average, 95% of the reads were aligned, out of which 72.95% were uniquely mapped to the reference (Supplementary Table S1). Counting of reads yielded a total of 30.821 genes with at least 10 read counts across the samples. After normalization by CPM, there were 19,784 genes detected with at least one CPM in at least three samples.

### 3.2 Polysome-recruited genes are differentially expressed throughout the cardiomyogenic differentiation

First, principal component analysis (PCA) was carried out to check the variability between the biological conditions in both experimental settings: the polysome-bound and ribosome-free fractions (Supplementary Figure S1B). Both PCAs show the expected clustering according to the different time points D0, D1, D4, D9, and D15. Additionally, the PCA already indicates a similarity of the expression profile between the initial developmental stages D0 and D1, the interesting distinction of D4, and the similarity between the end stages of D9 and D15 in accordance with the cardiomyogenic process.

Since we were interested in the genes that were recruited to the polysomes due to the possibility of translation control, we investigated the differential expression in the polysome-bound fractions, considering the pluripotent stage D0 as a control against all the other days of differentiation. The results were filtered to maintain only significantly expressed genes, considering the threshold of log2FoldChange ≥2, log2Foldchange ≤ −2, and adjusted *p*-value (padj) ≤ 0.05 and then separated by gene biotype (Supplementary Figures S1C,D).

### 3.3 Dynamic 3′ UTR length preference is associated to polysome recruitment throughout hESC cardiomyogenic differentiation

**FIGURE 1:**
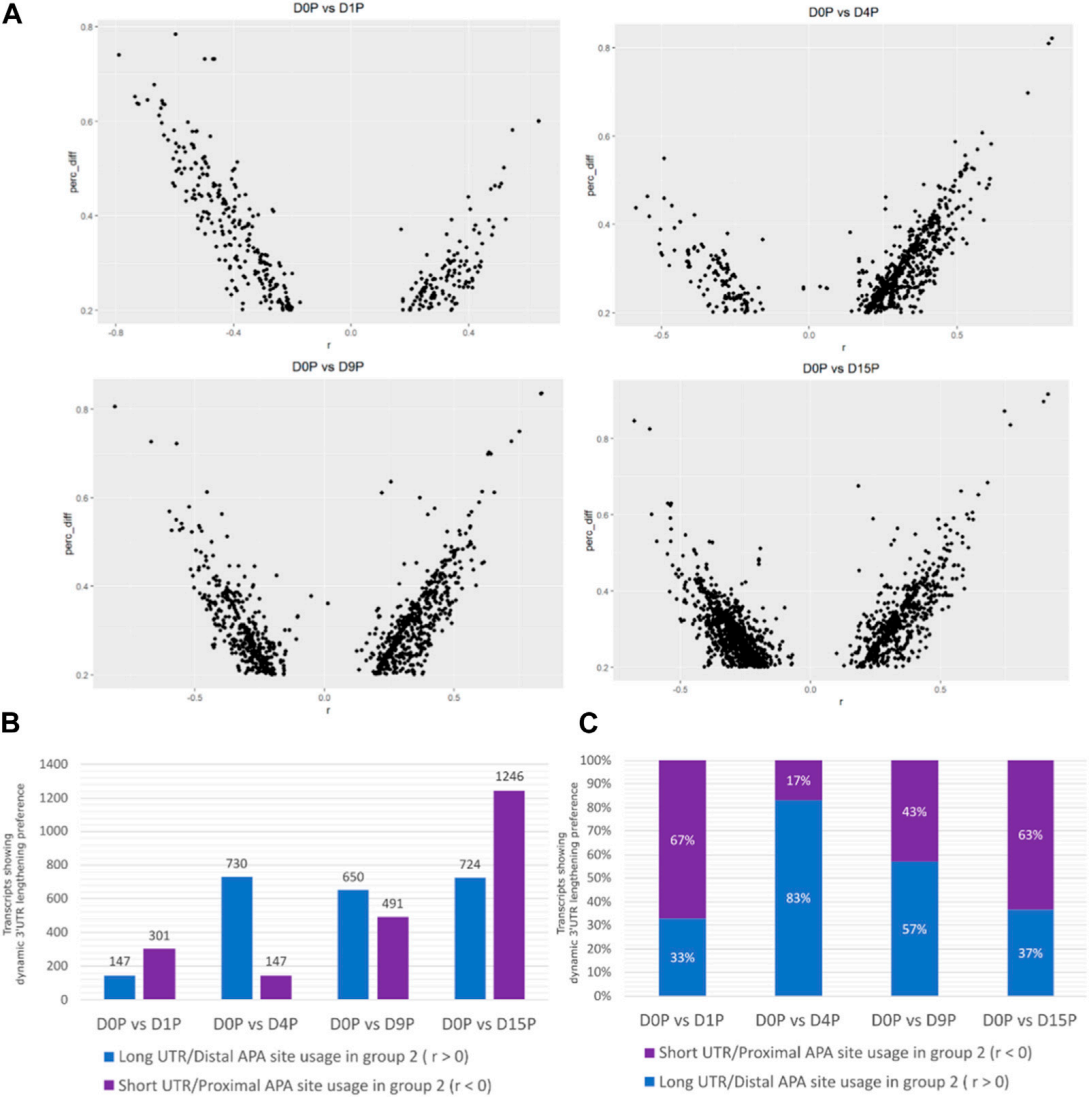
Dynamic changes of 3′ UTR lengthening and modulated APA site usage are seen during hESC differentiation to cardiomyocytes. **(A)** Transcripts with significant differential dynamic 3′ UTR length in polysomal fractions of D15. **(B)** Absolute quantity of transcripts. **(C)** Proportion of transcripts presenting dynamic 3′ UTR lengthening and shortening in polysomal fractions during cardiomyogenic differentiation, separated by day of differentiation and preference for 3′ UTR size.

Alternative polyadenylated transcripts were detected in all the days of cardiomyogenic differentiation (Figure 1A). The preference for distal or proximal polyadenylation sites was assessed, and surprisingly, the preference for long or short 3′ UTRs was different between the days (Figures 1A, B). Alternative polyadenylated transcripts were preferably expressed with shorter 3′ UTR on D1P (D1 polysome-bound) and D15P, while preference for longer 3′ UTR expression was observed on D4P and D9P. The most significant preference difference is observed on D4P, with the lowest number of transcripts favoring short 3′ UTR lengths throughout cardiomyogenesis, totaling 147 transcripts, while simultaneously exhibiting the highest number of transcripts favoring longer 3′ UTR lengths, with 730 APA isoforms. From D4P onwards, the quantity of preferred short 3′ UTR transcripts increases, reaching its peak on D15P, with 1,246 isoforms with proximal APA sites. Interestingly, proportionally, D1P and D15P are similar in their preference for proximal and distal sites, with 67% short isoforms in D1P and 63% in D15P. Proportionally, D4P is also the time point where there is a bigger difference in preference for the APA site, with only a 17% preference for short 3′ UTR and 83% for long 3′ UTR (Figure 1C).

**FIGURE 2:**
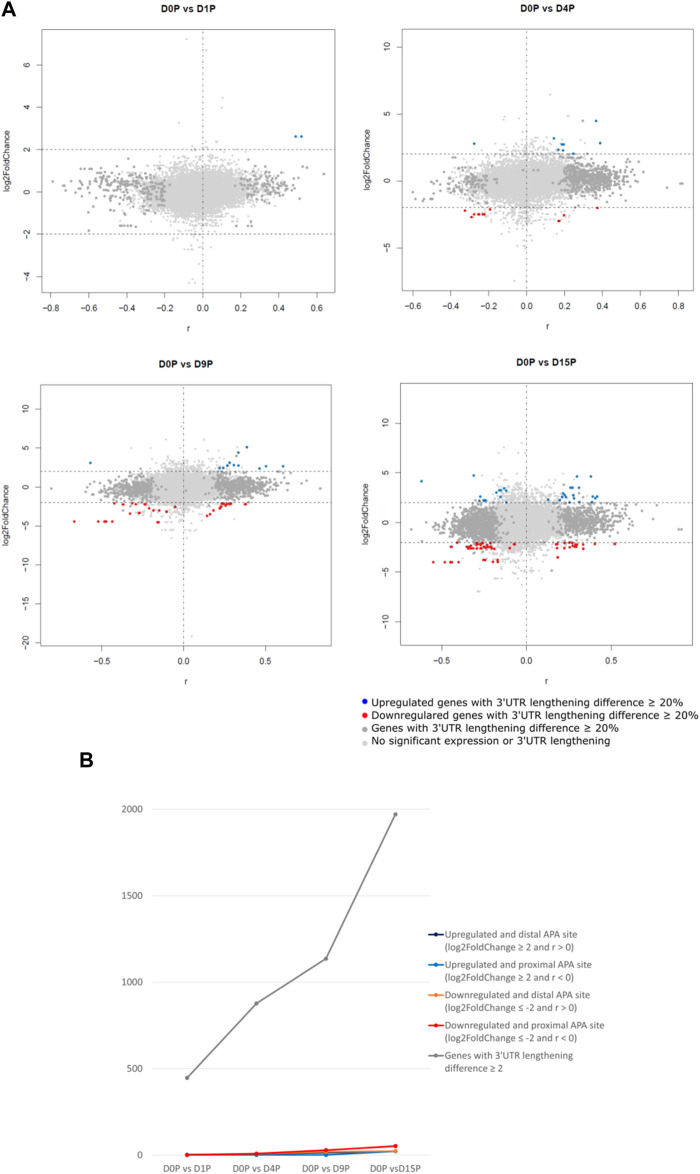
Differential expression is weakly associated with dynamic 3′ UTR length throughout hESC cardiomyogenic differentiation. **(A)** Differentially expressed polysome-bound transcripts show dynamic 3′ UTR lengthening during hESC differentiation to cardiomyocytes. **(B)** Quantification of the differentially expressed polysome-bound transcripts with dynamic 3′ UTR lengthening during hESC differentiation to cardiomyocytes.

We assessed which of these transcripts with dynamic 3′ UTR lengthening was also simultaneously differentially expressed. Curiously, the vast majority of transcripts that showed dynamic 3′ UTR lengthening were not simultaneously differentially expressed (Figure 2A). Interestingly, there is a steeper increase of dynamic 3′ UTR changes in comparison to the increase of the differential expression during cardiomyogenesis (Figure 2B). In D1P, only 2 out of 446 dynamic 3′ UTR transcripts were differentially expressed. Overall, the number of differentially expressed and dynamic 3′ UTR-lengthened transcripts increased with the ongoing cardiomyogenesis. In D4P, 877 genes showed significant dynamic 3′ UTR lengthening, out of which 9 were upregulated and 17 were downregulated, which leaves 851 transcripts showing only dynamic 3′ UTR length without differential expression. In D9P, of 1,136 genes with significant 3′ UTR lengthening, 16 were upregulated and 48 were downregulated, leaving 1,072 transcripts exclusively with dynamic 3′ UTR. Last, D15P showed 1,971 genes with significant 3′ UTR lengthening, out of which only 45 were upregulated and 75 were downregulated. Nonetheless, most genes that showed changes of 3′ UTR length were downregulated in all the differentiation days: 17 in D4P, 48 in D9P, and 75 in D15P, except for D1P, with no alternative polyadenylated and downregulated transcripts (Table 1).

**TABLE 1:** Number of differentially expressed and dynamic 3’ UTR lengthening transcripts in each comparison during cardiomyogenic differentiation, separated by the preference of the proximal or distal APA site.

	Upregulated distal APA site log2FC ≥ 2 r > 0	Upregulated proximal APA site log2FC ≥ 2 r < 0	Downregulated distal APA site log2FC ≤ −2 r > 0	Downregulated proximal APA site log2FC ≤ −2 r < 0	Genes with only 3′ UTR lengthening difference ≥0.2	No significant expression or lengthening
D0P vs. D1P	2	0	0	0	446	23,036
D0P vs. D4P	8	1	8	9	877	24,458
D0P vs. D9P	15	1	19	29	1,136	22,834
D0P vs. D15P	23	22	23	52	1,971	24,571

**FIGURE 3:**
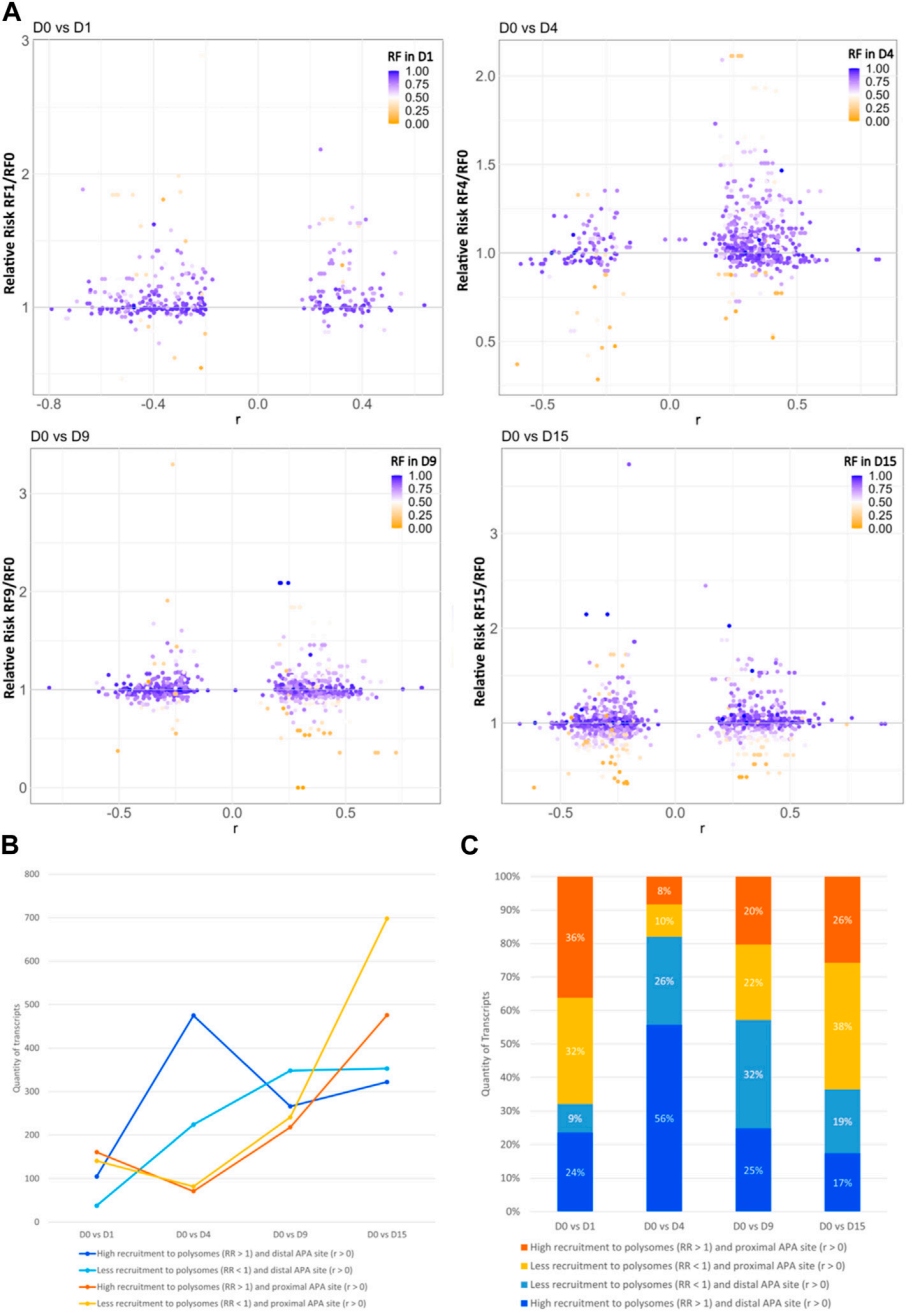
Dynamic 3′ UTR changes are associated with the recruitment of polysomes during hESC differentiation to cardiomyocytes. **(A)** Distribution of the RR of polysome recruitment according to the Pearson correlation coefficient (r) for the preference of 3′ UTR length; color maps of the RF of polysomal recruitment on each day of differentiation. **(B)** Number and **(C)** proportion of transcripts according to the RR of greater recruitment to polysomes in the day of differentiation (>1) or lower recruitment (<1) and the preference for distal APA site (r > 0) or proximal APA site (r < 0).

Since most of the genes that showed dynamic 3′ UTR lengthening were not differentially expressed, we assessed whether the dynamic 3′ UTR lengths were associated with the recruitment of these transcripts to the polysomal complexes. For this, we calculated the RF of polysomal fraction recruitment for each time point and the RR of polysomal recruitment in relation to D0, considering the changes in dynamic APA for each day of differentiation (see Materials and methods). The distribution of the RR of the recruitment of transcripts to polysomal fractions looks homogenous between the distal and proximal APA sites in each day of differentiation compared to D0P, except on D4P, where there is a higher concentration of transcripts highly recruited to polysomes and with a preference for distal APA sites (Figure 3A). When we look at the number of transcripts that are recruited and have a preference for distal/proximal APA sites, the differences are clearer (Figures 3B, C; Table 2). Compared to D0, on D1P, there is a slightly higher concentration of transcripts that show RR > 1 of being recruited to polysomes simultaneously with a preference for short 3′ UTR. Significantly, on D4P, there is a much higher concentration of transcripts with RR > 1 being recruited to polysomes concomitant to the preference for long 3′ UTR. Even though the RR being recruited to polysomes on D15P (with respect to D0P) is higher than 1, with some genes even surpassing RR > 2 (two times the risk of being recruited to D15P), there are more genes that show low RF recruitment to polysomal fraction. In addition, on D15P, there is the highest number of transcripts that are preferably recruited to polysomes in D0P, coinciding with the preference for short 3′ UTR or proximal APA sites (Figures 3B, C; Table 2). Last, we observed that recruitment to polysomes with a preference for proximal APA sites increases together throughout cardiomyogenesis. Nonetheless, transcripts recruited to polysomes with a preference for distal APA sites reach their peak on D4P and stabilize after D9P. A similar pattern of stabilization after D9P is seen in the transcripts that are recruited less to polysomes and prefer distal APA sites. It is possible to notice these two trends of recruitment of short 3′ UTR transcripts to polysomes increasing during differentiation, while recruitment of long 3′ UTR transcripts decreases and stabilizes during differentiation.

**TABLE 2:** Quantity of transcripts with dynamic 3′ UTR lengthening that are recruited into the polysomes on the day of differentiation or recruited to D0P.

	*High recruitment to polysomes (RR > 1) and distal APA site (r > 0)*	*High recruitment to polysomes (RR > 1) and proximal APA site (r > 0)*	*Less recruitment to polysomes (RR < 1) and distal APA site (r > 0)*	*Less recruitment to polysomes (RR < 1) and proximal APA site (r > 0)*	*Total transcripts*
D0 vs. D1	105 (24%)	161 (36%)	38 (9%)	141 (32%)	445
D0 vs. D4	475 (56%)	71 (8%)	224 (26%)	82 (10%)	852
D0 vs. D9	266 (25%)	218 (20%)	348 (32%)	241 (22%)	1,073
D0 vs. D15	322 (17%)	476 (26%)	353 (19%)	698 (38%)	1,849

These findings collectively suggest that the preferences for specific 3′ UTR lengths are associated with the recruitment of these transcripts from polysomes. This implies that post-transcriptional regulatory processes during cardiomyogenic differentiation may be influenced by alterations in APA site selection and 3′ UTR length preference, irrespective of any differential gene expression.

**FIGURE 4:**
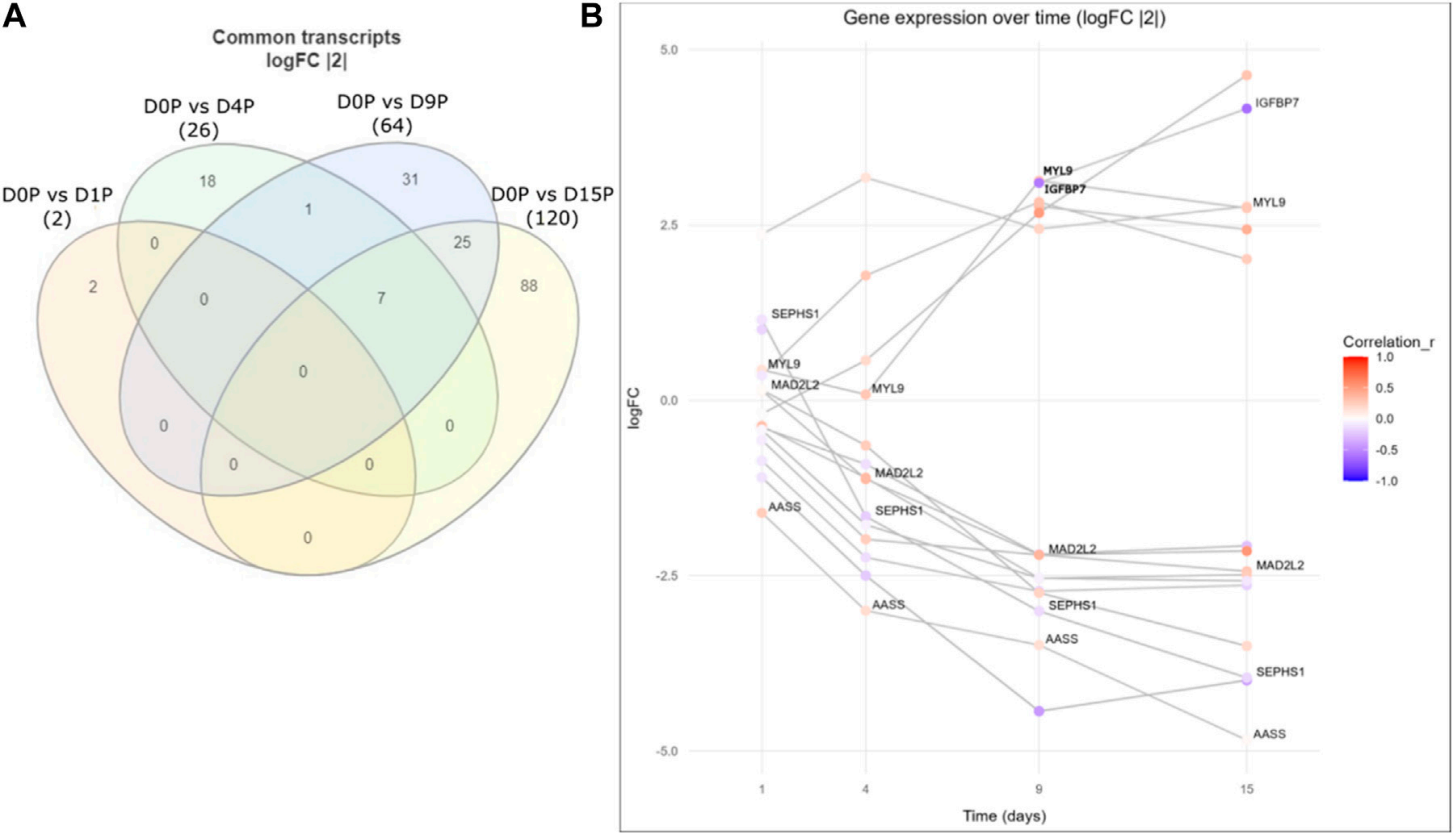
Common differentially expressed genes with dynamic 3′ UTR changes throughout the cardiomyogenesis of hESCs. **(A)** Venn diagram of common dynamic 3′ UTR lengthened transcripts and simultaneously differentially expressed genes with log2FoldChange |2|. **(B)** Gene expression over time of the same common differentially expressed genes with dynamic 3′ UTR length changes along with log2FoldChange values of |2|. Preference for 3′ UTR lengths is shown through the Pearson coefficient (r).

Finally, we assessed whether there were any common genes throughout all the days of the cardiomyogenesis process that were changing their preference of 3′ UTR length and fold change of expression depending on the day of differentiation. To investigate this, we created a Venn diagram with all the transcripts that showed dynamic 3′ UTR lengthening and were simultaneously differentially expressed in all time-point comparisons (Figure 4; Table 3). We set up a log2FoldChange cutoff of |2| to find more common genes that might have a lower expression. Then, we plotted the transcripts together with both their Pearson correlation coefficient for the preference of 3′ UTR length and their log2FoldChange, which were interconnected through the different time points. The result showed that many genes behave differently during the cardiomyogenesis process in terms of varied changes of 3′ UTR length and expression. Interestingly, two different trends were observed: one group of genes increased their expression throughout cardiomyogenesis, and another group decreased their expression until the final stages of differentiation. Examples of genes that increased expression were *IGFBP7* and *MYL9*, whereas genes that decreased their expression were *MAD2L2*, *SEPHS1*, and *AASS*. Concomitantly, changes in the preference of APA sites were also observed. The increased-expression group of genes also tended to show a preference for longer 3′ UTRs, while the decreasing-expression group tended to show a preference for shorter 3′ UTRs (Figure 4; Table 3). The gene *SEPHS1* was upregulated in D1P and changed to being downregulated in the subsequent days D4P, D9P, and D15P, while its APA isoforms showed a preference for short 3′ UTR during all time points. Similarly, *MAD2L2* also had the same differential expression pattern, upregulated only in D1P and downregulated in the other days, but with the contrary preference of long 3′ UTR during all time points. *IGFBP7* was only differentially expressed on D9 and D15, being preferable with short 3′ UTR on both days. *MYL9* showed a preference for long 3′ UTR in all time points, as well as *AASS* (Figure 4; Table 3).

**TABLE 3:** Values of log2FoldChange and Pearson correlation coefficient (r) of the common differentially expressed genes with dynamic 3′ UTR lengthening throughout all the days of cardiomyogenesis from hESCs.

Transcript ID	Gene ID	Gene name	r D1	log2FC D1	r D4	log2FC D4	r D9	log2FC D9	r D15	log2FCD15
ENST00000681387	ENSG00000008311	*AASS*	0.26	−1.60	0.17	−3.00	0.16	−3.49	0.03	−4.84
ENST00000413016	ENSG00000106992	*AK1*	0.07	−0.19	0.27	0.57	0.61	2.67	0.38	4.63
ENST00000550143	ENSG00000106992	*AK1*	0.00	−0.19	0.19	0.57	0.50	2.67	0.30	4.63
ENST00000375320	ENSG00000118137	*APOA1*	0.02	2.37	0.14	3.17	0.22	2.44	0.23	2.76
ENST00000677208	ENSG00000109861	CTSC	−0.03	−0.42	−0.05	−1.77	−0.05	−2.53	−0.10	−2.57
ENST00000678520	ENSG00000109861	CTSC	−0.03	−0.42	−0.05	−1.77	−0.05	−2.53	−0.10	−2.57
ENST00000679224	ENSG00000109861	CTSC	−0.03	−0.42	−0.05	−1.77	−0.05	−2.53	−0.10	−2.57
ENST00000465908	ENSG00000105993	DNAJB6	−0.08	−0.56	0.25	−1.98	0.38	−2.20	0.52	−2.15
ENST00000634080	ENSG00000105993	DNAJB6	−0.08	−0.56	0.25	−1.98	0.38	−2.20	0.52	−2.15
ENST00000592290	ENSG00000089327	FXYD5	0.31	−0.37	0.35	−1.10	0.23	−2.54	0.26	−2.49
ENST00000333762	ENSG00000184897	H1-10	−0.18	1.01	#N/A	#N/A	0.34	2.76	0.41	2.44
ENST00000328221	ENSG00000185885	IFITM1	−0.14	−1.10	−0.28	−2.50	−0.52	−4.43	−0.48	−3.99
ENST00000679380	ENSG00000185885	IFITM1	−0.13	−1.10	−0.25	−2.50	−0.47	−4.43	−0.44	−3.99
ENST00000680588	ENSG00000185885	IFITM1	−0.13	−1.10	−0.25	−2.50	−0.48	−4.43	−0.45	−3.99
ENST00000680696	ENSG00000185885	IFITM1	−0.14	−1.10	−0.23	−2.50	−0.67	−4.43	−0.55	−3.99
ENST00000680938	ENSG00000185885	IFITM1	−0.13	−1.10	−0.25	−2.50	−0.48	−4.43	−0.45	−3.99
ENST00000681426	ENSG00000185885	IFITM1	−0.11	−1.10	−0.22	−2.50	−0.44	−4.43	−0.40	−3.99
ENST00000512512	ENSG00000163453	IGFBP7	#N/A	#N/A	#N/A	#N/A	−0.57	3.10	−0.62	4.16
ENST00000376672	ENSG00000116670	MAD2L2	0.10	0.14	0.44	−1.12	0.28	−2.20	0.29	−2.44
ENST00000376692	ENSG00000116670	MAD2L2	0.09	0.14	0.39	−1.12	0.26	−2.20	0.29	−2.44
ENST00000279022	ENSG00000101335	MYL9	0.15	0.44	0.28	0.08	0.28	3.13	0.28	2.74
ENST00000346786	ENSG00000101335	MYL9	0.15	0.44	0.28	0.08	0.28	3.13	0.28	2.74
ENST00000282412	ENSG00000138032	PPM1B	−0.10	−0.86	−0.14	−2.24	−0.21	−2.72	−0.19	−2.64
ENST00000327347	ENSG00000086475	SEPHS1	−0.12	1.15	−0.21	−1.65	−0.15	−3.01	−0.17	−3.95
ENST00000378614	ENSG00000086475	SEPHS1	−0.16	1.15	−0.26	−1.65	−0.18	−3.01	−0.20	−3.95
ENST00000545675	ENSG00000086475	SEPHS1	−0.12	1.15	−0.21	−1.65	−0.15	−3.01	−0.17	−3.95
ENST00000358572	ENSG00000198498	TMA16	−0.20	−0.39	−0.16	−0.91	−0.29	−2.20	−0.29	−2.07
ENST00000509657	ENSG00000198498	TMA16	−0.18	−0.39	−0.13	−0.91	−0.23	−2.20	−0.26	−2.07
ENST00000513272	ENSG00000198498	TMA16	−0.18	−0.39	−0.13	−0.91	−0.23	−2.20	−0.26	−2.07
ENST00000259818	ENSG00000137285	TUBB2B	0.02	0.15	0.26	−0.64	0.22	−2.74	0.18	−3.50
ENST00000473006	ENSG00000137285	TUBB2B	0.02	0.15	0.26	−0.64	0.22	−2.74	0.18	−3.50
ENST00000681707	ENSG00000137285	TUBB2B	0.02	0.15	0.26	−0.64	0.22	−2.74	0.18	−3.50
ENST00000598676	ENSG00000131116	ZNF428	−0.10	0.36	0.29	1.78	0.31	2.82	0.25	2.02
ENST00000681387	ENSG00000008311	AASS	0.26	−1.60	0.17	−3.00	0.16	−3.49	0.03	−4.84
ENST00000413016	ENSG00000106992	AK1	0.07	−0.19	0.27	0.57	0.61	2.67	0.38	4.63
ENST00000550143	ENSG00000106992	AK1	0.00	−0.19	0.19	0.57	0.50	2.67	0.30	4.63
ENST00000375320	ENSG00000118137	APOA1	0.02	2.37	0.14	3.17	0.22	2.44	0.23	2.76
ENST00000677208	ENSG00000109861	CTSC	−0.03	−0.42	−0.05	−1.77	−0.05	−2.53	−0.10	−2.57
ENST00000678520	ENSG00000109861	CTSC	−0.03	−0.42	−0.05	−1.77	−0.05	−2.53	−0.10	−2.57
ENST00000679224	ENSG00000109861	CTSC	−0.03	−0.42	−0.05	−1.77	−0.05	−2.53	−0.10	−2.57
ENST00000465908	ENSG00000105993	DNAJB6	−0.08	−0.56	0.25	−1.98	0.38	−2.20	0.52	−2.15
ENST00000634080	ENSG00000105993	DNAJB6	−0.08	−0.56	0.25	−1.98	0.38	−2.20	0.52	−2.15
ENST00000592290	ENSG00000089327	FXYD5	0.31	−0.37	0.35	−1.10	0.23	−2.54	0.26	−2.49
ENST00000333762	ENSG00000184897	H1-10	−0.18	1.01	#N/A	#N/A	0.34	2.76	0.41	2.44
ENST00000328221	ENSG00000185885	IFITM1	−0.14	−1.10	−0.28	−2.50	−0.52	−4.43	−0.48	−3.99
ENST00000679380	ENSG00000185885	IFITM1	−0.13	−1.10	−0.25	−2.50	−0.47	−4.43	−0.44	−3.99
ENST00000680588	ENSG00000185885	IFITM1	−0.13	−1.10	−0.25	−2.50	−0.48	−4.43	−0.45	−3.99
ENST00000680696	ENSG00000185885	IFITM1	−0.14	−1.10	−0.23	−2.50	−0.67	−4.43	−0.55	−3.99
ENST00000680938	ENSG00000185885	IFITM1	−0.13	−1.10	−0.25	−2.50	−0.48	−4.43	−0.45	−3.99
ENST00000681426	ENSG00000185885	IFITM1	−0.11	−1.10	−0.22	−2.50	−0.44	−4.43	−0.40	−3.99
ENST00000512512	ENSG00000163453	IGFBP7	#N/A	#N/A	#N/A	#N/A	−0.57	3.10	−0.62	4.16
ENST00000376672	ENSG00000116670	MAD2L2	0.10	0.14	0.44	−1.12	0.28	−2.20	0.29	−2.44
ENST00000376692	ENSG00000116670	MAD2L2	0.09	0.14	0.39	−1.12	0.26	−2.20	0.29	−2.44

In summary, these findings demonstrate the dynamic nature of the cardiomyogenesis process. In terms of the expression of genes and their alternative APA isoforms, the same set of genes can show different outcomes throughout the differentiation process. The dynamic regulation of 3′ UTR lengths and the expression of isoforms is relevant and unique for each time point individually and does not reflect only the differentiated cardiomyocyte state since the beginning of the differentiation process.

### 3.4 Lengthened and shortened 3′ UTRs are the targets of miRNAs expressed during the cardiomyogenic differentiation of hESCs

To assess whether the alternative polyadenylated transcripts and differentially expressed genes were being regulated by microRNAs targeting their modulated 3′ UTR regions, we first selected the expressed miRNAs during hESC cardiomyogenic differentiation (Garate et al., 2018). Since we were interested in discovering if additional miRNA target sites were appearing due to the lengthening of the 3′ UTRs and considering that miRNAs preferably connected to the 3′ UTR position of the transcripts, mostly leading to their inactivation (Krol et al., 2010), we predicted miRNA targeting specifically on the sequences of the expressed 3′ UTRs of our data. We found the following four possible modulations of miRNA and gene expression: coordinated (upregulated miRNA and target gene; downregulated miRNA and target gene) and uncoordinated (upregulated miRNA and downregulated target gene; downregulated miRNA and upregulated target genes) (Supplementary Figure S2A). Most miRNA–gene target pairs were coordinated downregulated, with 106 miRNA–gene pairs in D4, 229 in D9, and 330 in D15. The lowest number of miRNA–gene pairs was coordinated upregulated, accounting for 12 miRNA–gene pairs in D4, 42 in D9, and 56 in D15. The uncoordinated modulation of downregulated miRNA and the upregulated gene target obtained 19 miRNA–gene pairs in D4, 60 in D9, and 174 in D15. Last, upregulated miRNAs and downregulated gene targets accounted for 75 miRNA–gene pairs in D4, 115 in D9, and 186 in D15. As mentioned, most targeted genes were coordinated downregulated. Since miRNA regulation can target more than one gene, and accordingly, one gene can be targeted by more than one miRNA, we focused on the uncoordinated results with upregulated miRNAs and downregulated targets.

Validation of the gene–miRNA target pairs was corroborated using different *in silico* sources, investigating the relevance of these targets for hESC differentiation and the confirmation of the miRNA-binding site on the 3′ UTR of the target. Some genes from cardiomyocyte differentiation day 15 were selected due to their relevant connection through miRNA targets, which are *FGFR1, SEPHS1,* and *ALKBH5*. All are differentially expressed in D15, with *FGFR1* showing downregulation with a log2FoldChange of −2.06, *SEPHS1* also being downregulated with a log2FoldChange of −3.95, and *ALKBH5* being upregulated with a log2FoldChange of 2.03. On the database miRWalk (Sticht et al., 2018), the prediction coincides with our own, as it suggests that the miRNA hsa-let-7e-5p is expected to target the gene *SEPHS1*, specifically within the 3′ UTR position. In our results, this miRNA is upregulated with a log2FoldChange of 5.34 (Table 4, Supplementary Figure S2B). The genes *FGFR1* and *ALKBH5* shared a low-expressed miRNA target, namely, hsa-mir-197-3p. Even though this miRNA is upregulated in D15 with a log2FoldChange of 1.97, lower than the threshold of |2| (Table 4), an additional *in silico* analysis on the experimentally supported database ENCORI showed that both genes share the same miRNA in a competing endogenous RNA (ceRNA) regulation manner (Supplementary Figure S2C).

**TABLE 4:** Differentially expressed genes *SEPHS1,* *FGFR1,* and *ALKBH5* with alternative polyadenylated transcripts targeted on the 3’ UTR position by differentially expressed miRNAs on day 15 of differentiation of hESCs to cardiomyocytes.

Target ID	microRNA	log2FC miRNA	Padj miRNA	Target name	Target transcript ID	Percentage difference	r	Padj UTR	log2FC DEG	Padj DEG
ENSG00000086475	hsa-let-7e-5p	5.34	0.00	*SEPHS1*	ENST00000327347	0.30	−0.17	0.00	−3.95	0.00
ENSG00000086475	hsa-let-7e-5p	5.34	0.00	*SEPHS1*	ENST00000545675	0.30	−0.17	0.00	−3.95	0.00
ENSG00000086475	hsa-miR-302d-3p	−7.08	0.00	*SEPHS1*	ENST00000327347	0.30	−0.17	0.00	−3.95	0.00
ENSG00000086475	hsa-miR-302d-3p	−7.08	0.00	*SEPHS1*	ENST00000545675	0.30	−0.17	0.00	−3.95	0.00
ENSG00000077782	hsa-miR-197-3p	1.97	0.00	*FGFR1*	ENST00000531196	0.30	0.27	0.00	−2.06	0.00
ENSG00000077782	hsa-miR-197-3p	1.97	0.00	*FGFR1*	ENST00000397108	0.25	0.22	0.00	−2.06	0.00
ENSG00000091542	hsa-miR-197-3p	1.97	0.00	*ALKBH5*	ENST00000399138	0.26	−0.12	0.03	2.03	0.00

### 3.5 Dynamic 3′ UTR lengthening is involved in the gene regulatory networks during the cardiomyogenic differentiation of hESCs

**FIGURE 5:**
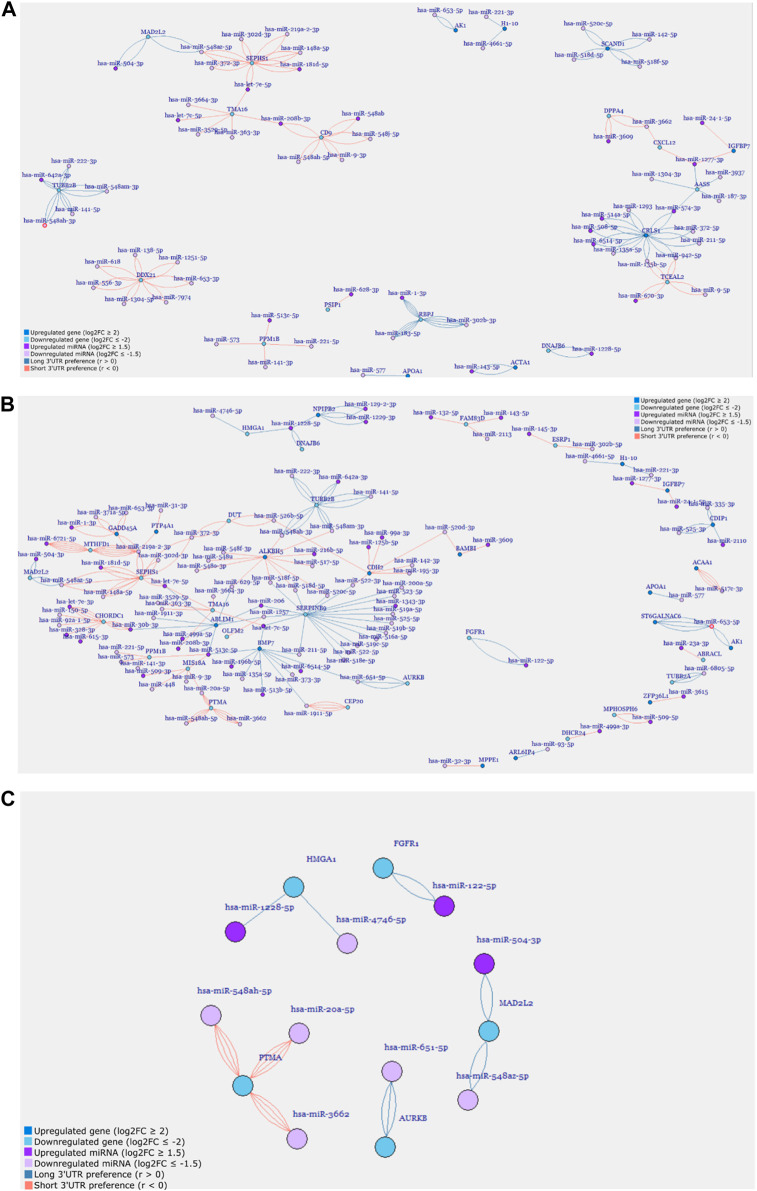
Gene regulatory networks of **(A)** D9P, **(B)** D15P, and **(C)** selected cell-cycle-related genes from the cardiomyogenic differentiation of hESC. Genes and miRNA are the vertices, and the transcripts are the edges.

Utilizing the miRNA target information on the 3′ UTR sites, we systematically constructed individual gene regulatory networks for the days D4, D9, and D15 of the cardiomyogenic differentiation process from hESCs (Supplementary Figure S3, Figures 5A–C). At the second time point, D1, there were insufficient genes available to construct a gene regulatory network, as already shown (Figure 1; Figure 2; Table 1). This approach allowed us to visualize the specific interactions between differentially expressed miRNAs and genes and how they interplay with alternative APA isoforms. Additionally, we could simultaneously examine the preference of the APA transcripts for longer or shorter 3′ UTRs, providing a comprehensive understanding of the regulatory landscape.

In the first network generated, D4P did not show any connections between genes (Supplementary Figure S3). Some genes showed many miRNA targets, such as *CNTNAP2*, which was targeted by six different miRNAs, but no other gene shared these miRNAs to make any connection. The gene *AASS* was targeted by only two other miRNAs; however, they were targeting six different APA isoforms each. Out of these six genes, only two genes had transcripts that showed a preference for short 3′ UTR, which are *SPP1* and *PMAIP1*, while the other four genes had transcripts that showed a preference for long 3′ UTR.

The network for D9P was bigger than that for the previous time point, with 105 miRNA and gene targets and 182 edges (Figure 5A, Supplementary Table S2). Interestingly, two subnetworks appeared, in which the genes are interconnected through their shared miRNA targets. One cluster is composed of *SEPHS1, MAD2L2, CD9, TMA16,* and *ACTA1*. The other cluster is composed of *IGFBP7, AASS, CXCL12, CRLS1, TCEAL2, TUBB2B,* and *DPPA4*. The gene AASS is present in the network D4P, and here, it shares miRNAs with two other genes in the cluster. Both clusters have genes whose APA isoforms show preferences for both short and long 3′ UTRs. These clusters connected by shared miRNAs are the first piece of evidence of competing endogenous RNA starting on D9 of the cardiomyogenic process.

When we look at the network for D15P, not only did the number of gene targets and miRNA increase but also the edges connecting them, which were 175 and 280, respectively (Supplementary Table S2; Figure 5B). We could visually notice that the second subnetwork seen in D9P is lost, whereas the first subnetwork increases the number of genes interconnected through shared miRNAs, forming a big regulatory network for D15P (Figure 5B). This is demonstrated by a gene that was present in the previously disassembled subnetwork and is now incorporated into the big network of D15P, which is *TUBB2B*. The gene *IGFBP7* also participated in the disassembled cluster; however, it was not incorporated into the network of D15P. There are still some genes that share miRNAs outside the big network, such as *HMGA1*, *DNAJB6, NPIPB2*, *AK1*, and *ST6GALNAC6*, but they do not form a second significant cluster such as the others seen in D9P (Figure 5A).

**FIGURE 6:**
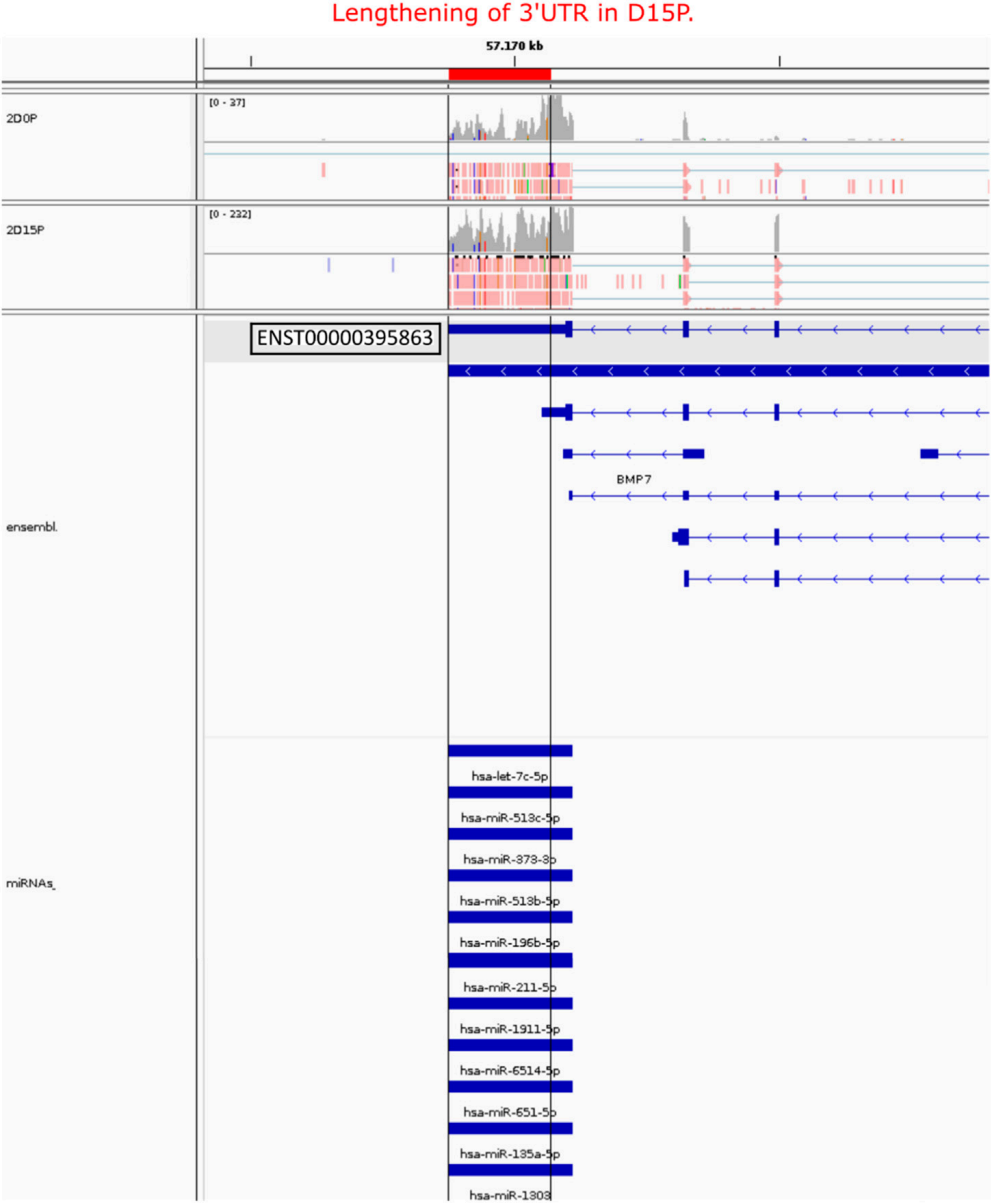
Visualization of the alignment of the BMP7 APA isoform, its 3′ UTR length, and its respective miRNA targets. Only the highlighted transcript ENST00000395863 was detected showing lengthening of 3′ UTR length in D15P compared to D0P. All miRNAs listed were targeting the lengthened 3′ UTR isoform on D15P. Gray vertical lines guide the lengthening difference of 3′ UTR length on D15P compared to D0P. At the bottom, the annotation of the transcript is represented in blue, in which boxes indicate exons and lines with arrows indicate introns and orientation in the genome. On the top, reads are represented in red with the read coverage on top. Only two replicates are exemplified in this figure, the second replicate of D0P and the second replicate of D15P.

After analyzing the regulatory network of D15P, some downregulated genes caught our attention regarding their known relationship to the cell cycle, including *FGFR1*, *MAD2L2*, *AURKB*, *HMGA1*, and *PTMA* (Figure 5C). The gene *CDC20* (cell division cycle 20) is related to the cell cycle, is downregulated, and shows dynamic 3′ UTR lengthening, but it did not appear in the regulatory network since it was not targeted by any expressed miRNA in our analysis (data not shown). Although they share miRNAs with other genes in the D15P network, such as *BMP7* and *ABLIM1*, they do not share common miRNAs between them. All of them show a preference for longer 3′ UTR, except *PTMA*, which shows a preference for short 3′ UTR. Visualization of the alignments in D15P for the gene *BMP7* in IGV made possible the identification of the dynamic 3′ UTR lengthening, the specific target miRNA on the isoform, and the coverage of reads that indicates the preference for long 3′ UTR usage in D15P (Figure 6).

These results suggest a possible multilayer regulatory interplay between differentially expressed genes, miRNAs, and the dynamics of 3′ UTR lengthening during cardiomyogenesis. Considering that these transcripts were identified and recruited by polysomes, altogether, these results suggest a complex post-transcriptional translational control during the differentiation of hESCs to cardiomyocytes.

## 4 Discussion

In this work, we analyzed hESC cardiomyogenic differentiation through a post-transcriptional perspective, investigating the dynamic 3′ UTR length change and possible regulation consequences in the polysomal transcriptome. Our results indicate that dynamic 3′ UTR length can influence post-transcriptional regulation through the availability of miRNA target sites during the differentiation of hESCs to cardiomyocytes.

During cardiomyogenesis, each time point of cardiomyogenesis shows a preference for either short or long 3′ UTR length, as compared to the pluripotent stage, reaching differentiated cardiomyocytes with a preference for short 3′ UTRs on D15P. Changes in 3′ UTR length have already been seen before during T-cell and hematopoietic differentiation (Sandberg et al., 2008; Gruber et al., 2014; Sommerkamp et al., 2020). In contrast with other works that show that pluripotent stem cells prefer longer 3′ UTRs in their differentiated state (Sommerkamp et al., 2021), our data indicate that cardiomyocytes in D15 of differentiation appear to preferably express short 3′ UTRs in comparison to the pluripotent stage in D0 of hESCs. Interestingly, the preference for short 3′ UTR is not a steady pattern seen throughout the whole differentiation process. Fluctuation of the preference of APA sites was seen from D1 of differentiation to the other, with the biggest change in cardiac mesodermal commitment on day 4, especially those transcripts that are simultaneously being recruited to polysomal complexes (D4P). The fourth day of differentiation is a crucial time point during hESC differentiation due to its commitment to cardiomyogenesis instead of other cell types (Loh et al., 2016).

Concomitantly, these transcripts are being regulated not only by differential expression but also by the recruitment to polysomal complexes. It was already seen that the cardiac stage of cardiomyogenesis shows regulatory aspects through the recruitment of polysomes (Pereira et al., 2019), but here, we see that it is also associated with the dynamic 3′ UTR changes.

Changes in 3′ UTR length can impact transcript regulation by the gain or loss of regulatory target sites and consequently affect the processes in the cell and, eventually, the proteomic outcome (Gruber et al., 2014; Turner et al., 2018; Mayr, 2019; Sommerkamp et al., 2021). Even though we did not investigate the proteome outcome, the post-transcriptional regulation through polysome recruitment indicates translation control (Schwanhüusser et al., 2011; Tebaldi et al., 2012) throughout the stem cell differentiation process (SPANGENBERG et al., 2013; PEREIRA et al., 2018; ROBERT et al., 2020).

Moreover, APA regulation can change the 3′ UTR landscape of the cell (Mitschka and Mayr, 2022). Therefore, it is expected that hESC cardiomyogenesis differentiation is complexly orchestrated by various layers of regulation that include polysome recruitment as well as 3′ UTR lengthening or shortening. This confirms the importance of investigating the post-transcriptional aspects of the differentiation process instead of stopping at gene expression.

For the miRNA target analysis, we guaranteed that both miRNA and their gene target were expressed in the respective differentiation days, which is an essential step to ensure that the regulation found can indeed occur (van Dam et al., 2018). By thoroughly analyzing some of the differentially expressed genes that were targeted by miRNAs expressed during cardiomyogenic differentiation, we corroborated our target predictions on the position of the 3′ UTR of those transcripts on experimentally validated databases, such as miRWalk and ENCORI (J. H. Li et al., 2014; Sticht et al., 2018). In this manner, the gene *SEPHS1* was found downregulated on day 15 of cardiomyogenic differentiation, and it was targeted on the 3′ UTR by the upregulated microRNA hsa-let-7e-5p. The family of let-7 microRNAs is essential for cardiomyocytes derived from stem cells, as it is required for their correct maturation (Kuppusamy et al., 2015; Kumar et al., 2019). These miRNAs are also found highly expressed in hESCs, possibly playing a role in their self-renewal properties (Koh et al., 2010). In addition, *SEPHS1* was found to be targeted by miRNA hsa-mir-302d-3p, which is well known for playing a key role in the proliferation of human pluripotent stem cells, including the ones that derive from cardiomyocytes (H.-L. Li et al., 2016; Xu et al., 2019). The gene *SEPHS1* itself is essential for cardiomyogenesis derived from mouse ESC (Qiao et al., 2022), which also supports our findings. The interconnection of genes through the share of common miRNAs was evidenced by the pair of downregulated *FGFR1* and upregulated *ALKBH5* genes, both of which were targeted by the upregulated hsa-mir-197-3p of a log2FoldChange of 1.97. Even though this miRNA did not make the threshold of a log2FoldChange of |2| by 0.03, such a relationship between both genes was corroborated in the experimentally validated ENCORI database as a ceRNA regulatory relationship. The competing endogenous RNA is a unifying hypothesis, which suggests that the transcripts compete for the same pool of miRNAs due to shared target sites (Salmena et al., 2011). Supporting this hypothesis are the subnetworks that appear first in D9 and then in D15 through the connection of common miRNAs.

Since the downregulation of *FGFR1* is known to play an essential role in maintaining cardiomyocytes in their mature form and preventing entry into the cell cycle (Valussi et al., 2021), we investigated if there were more downregulated genes related to the cell cycle in our gene regulatory networks. It is known that differentiated cells and cardiomyocytes show low proliferation capability due to their inability to reenter the cell cycle (Mohamed et al., 2018; Gladka et al., 2023). Suppressed cell cycle genes were identified on the 15th day of cardiomyogenesis, which corresponds to the differentiated cardiomyocyte time points: *MAD2L2, AURKB, FGFR1, HMGA1, PTMA*, and *CDC20* (cell division cycle 20). Gene expression over time showed that *MAD2L2* and *PTMA* decreased expression over time. The activation of *CDC20* plays a role in cell cycle progression inclusive in cardiomyocytes (Yamada et al., 2011), and its low level of expression is related to the binucleation of cardiomyocytes (Milliron et al., 2019). Together with *MAD2L2* (mitotic arrest deficient 2-like), they are known to interact to modulate mitosis prior to anaphase and chromosome separation (Pfleger et al., 2001; Listovsky and Sale, 2013). Moreover, *MAD2L2* is necessary for maintaining pluripotency in embryonic stem cells (Pirouz et al., 2015; Rahjouei et al., 2017). As for the gene *AURKB* (also known as Aurora B), it is also a key regulator of mitosis, playing a role in cell division by aligning and segregating the chromosomes (Nigg, 2001; Honda et al., 2003). Apart from being an indicator of cell division, inclusive of cardiomyocytes (Wang et al., 2020), its marker is used to demonstrate cardiac regeneration (Fu et al., 2020).

The gene *HMGA1* encodes a key protein that permits the access of small binding proteins to the DNA, which consequently impacts gene expression regulation (Vignali and Marracci, 2020). Although abundantly expressed during embryogenesis and important to maintain stemness, it is found downregulated in adult differentiated tissues (Vignali and Marracci, 2020). In our gene regulatory network, *HMGA1* transcripts showed a preference for longer 3′ UTRs. Other works have already shown that an increase in mRNA of *HMGA1* is not correlated to increased protein, demonstrating post-transcriptional regulation due to the binding of regulatory elements within the 3′ UTR (Borrmann et al., 2001). A shortage of 3′ UTR of *HMGA1* increased its expression, which corroborates with our regulatory network finding where downregulated *HMGA1* transcripts showed a preference for longer 3′ UTR. Similarly, prothymosin alpha (*PTMA*) is important for the binding of transcription factors to DNA and is required for cell division (Piñeiro et al., 2000; Samara et al., 2017). The overexpression of *PTMA* in cardiomyocytes stimulated their proliferation through the increased expression of the cell cycle genes and improved cardiac regeneration; thus, it is a relevant gene for the treatment of myocardial diseases (Gladka et al., 2023). Finally, *FGFR1* plays an important role in hESC differentiation when the suppression of *FGFR1* and activation of *BMP7*, also seen in our D15 network, help the switch to the differentiation state during cardiomyogenesis (Parikh et al., 2015). In summary, these results align with previous studies that indicate that cardiomyocytes derived from various sources tend to be predominantly quiescent and experience cell cycle arrest (Karbassi et al., 2020).

While certain aspects of post-transcriptional gene expression regulation were not addressed in this study, it is important to note that APA regulation can exert an influence beyond the inhibitory actions of miRNAs. In addition to miRNAs, the involvement of RNA-binding proteins (RBPs), transcription factors (TFs), and other factors plays a crucial role in the post-transcriptional regulation and can, therefore, be affected by the 3′ UTR lengthening and shortening (CORBETT, 2018). The analysis of the isoform networks could be further refined by considering aspects such as RNA half-life (Furlan et al., 2021) and the inhibitory potential of individual miRNA molecules, which unfortunately could not be incorporated into the present work. Moreover, the exclusion of long noncoding RNAs (lncRNAs) from the gene regulation network means that we did not account for their potential role as miRNA sponges (Thomson and Dinger, 2016), which could significantly impact the inhibitory capabilities of miRNAs and further affect the post-transcriptional regulatory network of cardiomyogenic differentiation.

## 5 Conclusion

Our prior research has highlighted the significance of post-transcriptional regulation, emphasizing the role of both coding and non-coding transcripts bound to polysomes in the cardiomyogenic differentiation process (Pereira et al., 2018; Pereira et al., 2019). Building on this foundation, we now explored how APA-induced alterations in 3′ UTR length impact the post-transcriptional landscape in each of the cardiomyogenesis stages. Usage of APA sites can change based on cellular conditions and cell type (Sommerkamp et al., 2021), and here, we highlight that each stage has its own post-transcriptional APA modifications that are also associated with the recruitment of polysomes. Even though gene-regulatory networks are usually constructed with elements such as differentially expressed genes, transcription factors, and binding proteins (Parikh et al., 2015), here, we showed that a network based on dynamic 3′ UTR lengthening changes might support the discovery of miRNA target regulation. Hence, we look at another layer of post-transcriptional regulation during hESC differentiation to cardiomyocytes, one that is dependent on alternative isoforms even though it expresses the same number of genes.

Cardiomyogenic differentiation is a complex process involving the activation and repression of specific genes at different stages. Investigation of transcriptional regulation coupled to polysomal recruitment during the stem cell differentiation process is crucial for understanding the translational dynamics of gene expression in cardiomyogenesis. Together with the changing 3′ UTR lengthening landscape, we were able to observe the temporal dynamics of translation and identify the key genes involved in the differentiation process that are actively recruited into the translational machinery. Other studies also confirmed that APA can regulate stem cell behavior and function directly (Sommerkamp et al., 2021). Therefore, our work provides novel insights into the intricate post-transcriptional regulatory processes during cardiomyogenesis, where differentially expressed genes, differentially expressed miRNAs, and the dynamic lengthening of 3′ UTRs through APA all play a significant role.

This investigation holds promise for advancing our understanding of the post-transcriptional regulatory processes governing stem cell fate commitment, with implications for regenerative medicine and therapeutic strategies for cardiovascular diseases. Understanding the translation control of specific genes can guide the development of strategies to enhance or manipulate the differentiation process for therapeutic purposes.

While this work successfully identified 3′ UTR lengthening patterns in the RNA-seq data, it is crucial to acknowledge certain limitations. Specifically, the utilization of polyadenylation site sequencing (PAS-Seq) or RNA-seq with 3′ end enrichment techniques could offer a more refined and detailed understanding of the 3′ UTR landscape. Incorporating these methodologies in future investigations would enhance the precision and depth of the regulatory network analysis.

## Data Availability

The datasets presented in this study can be found in online repositories. The names of the repository/repositories and accession number(s) can be found at: https://www.ncbi.nlm.nih.gov/, SRP150416.
