# Supplementary figures and images for: Alternative polyadenylation and dynamic 3′ UTR length is associated with polysome recruitment throughout the cardiomyogenic differentiation of hESCs
Source: Front Mol Biosci. 2024 Feb 6;11:1336336. doi: 10.3389/fmolb.2024.1336336 (PMC10877728; doi:10.3389/fmolb.2024.1336336)

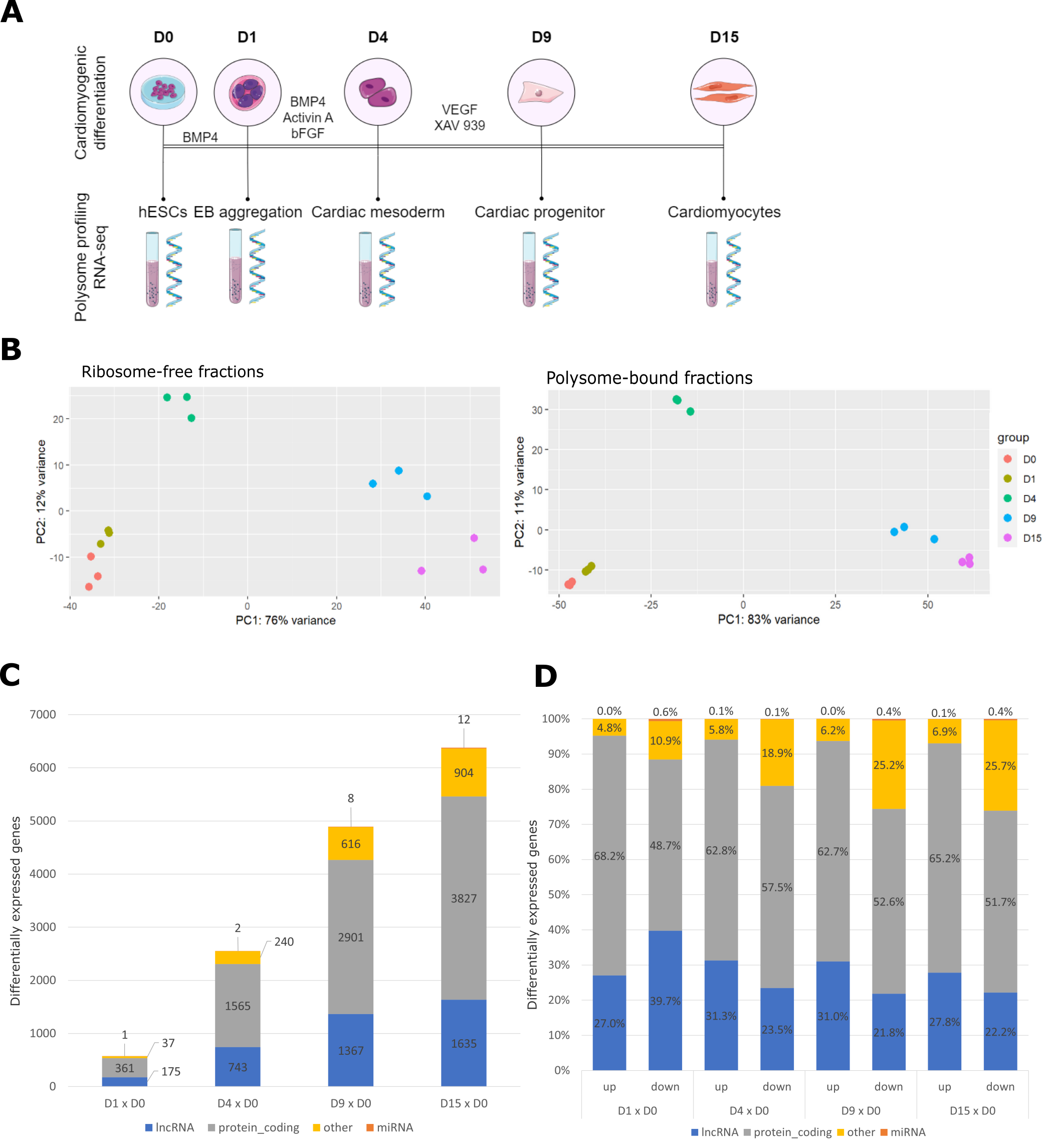

Supplement: Supplementary file 1 [file Image3.TIFF]

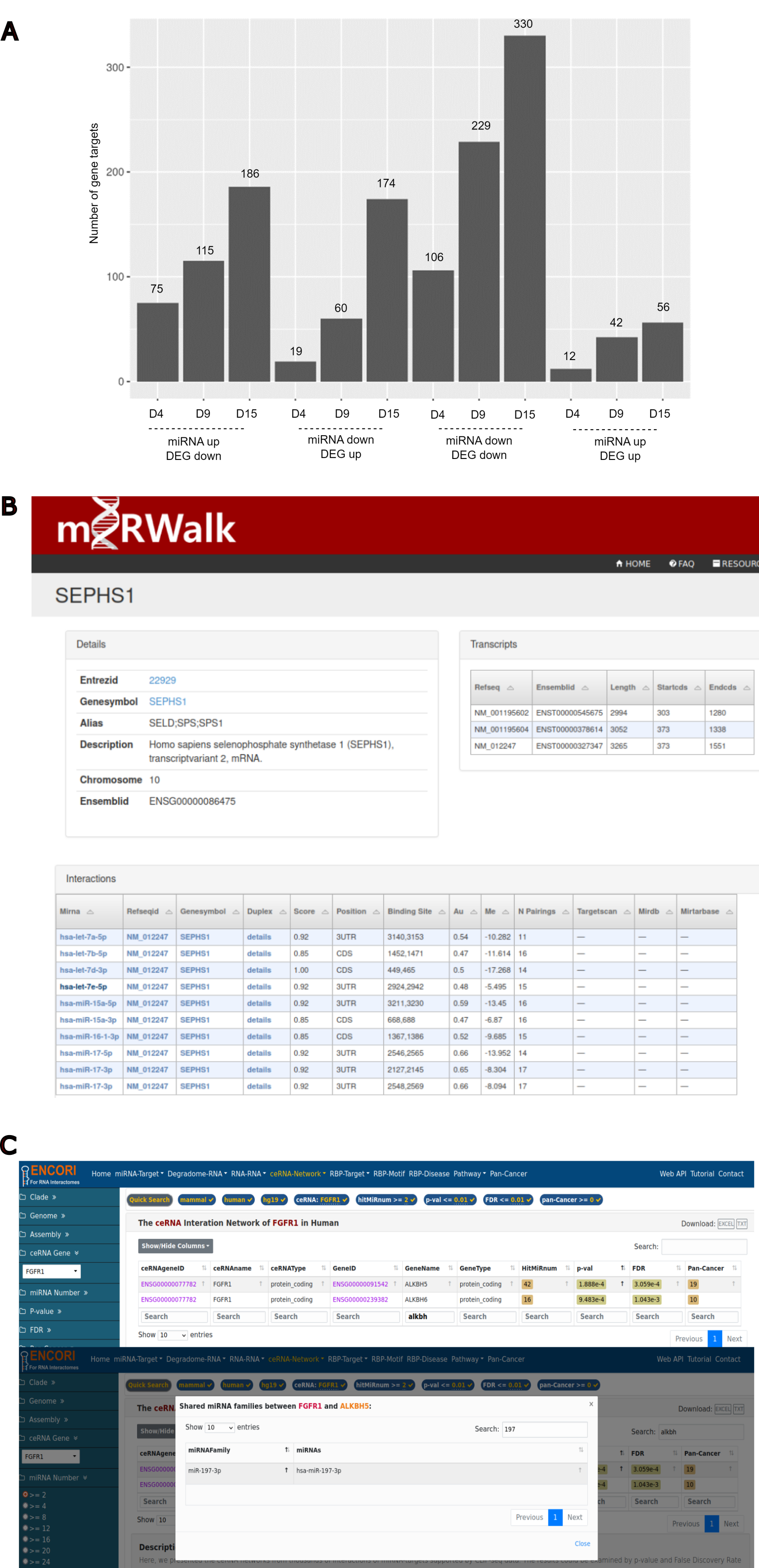

Supplement: Supplementary file 2 [file Image1.TIFF]

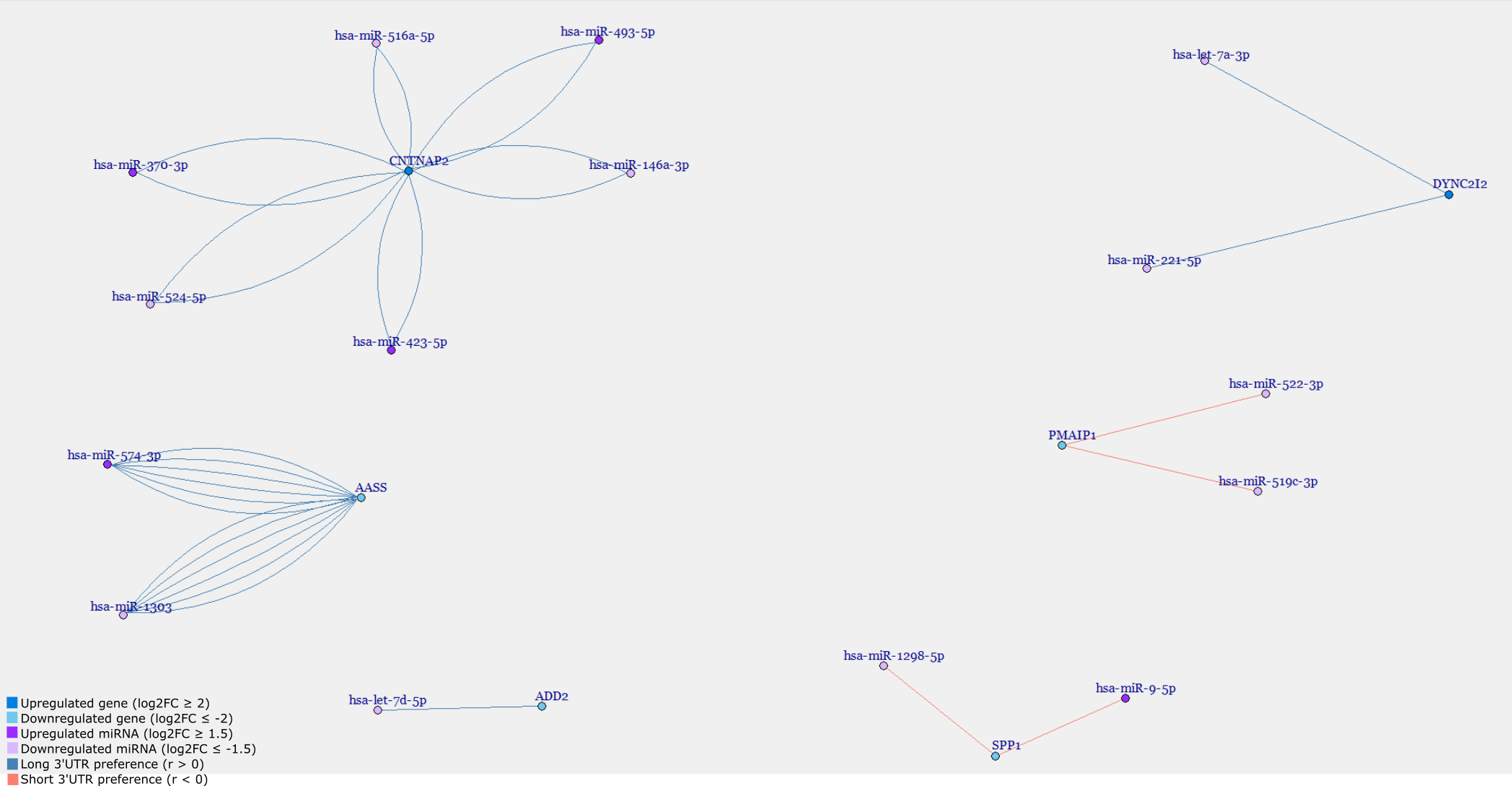

Supplement: Supplementary file 4 [file Image2.TIFF]

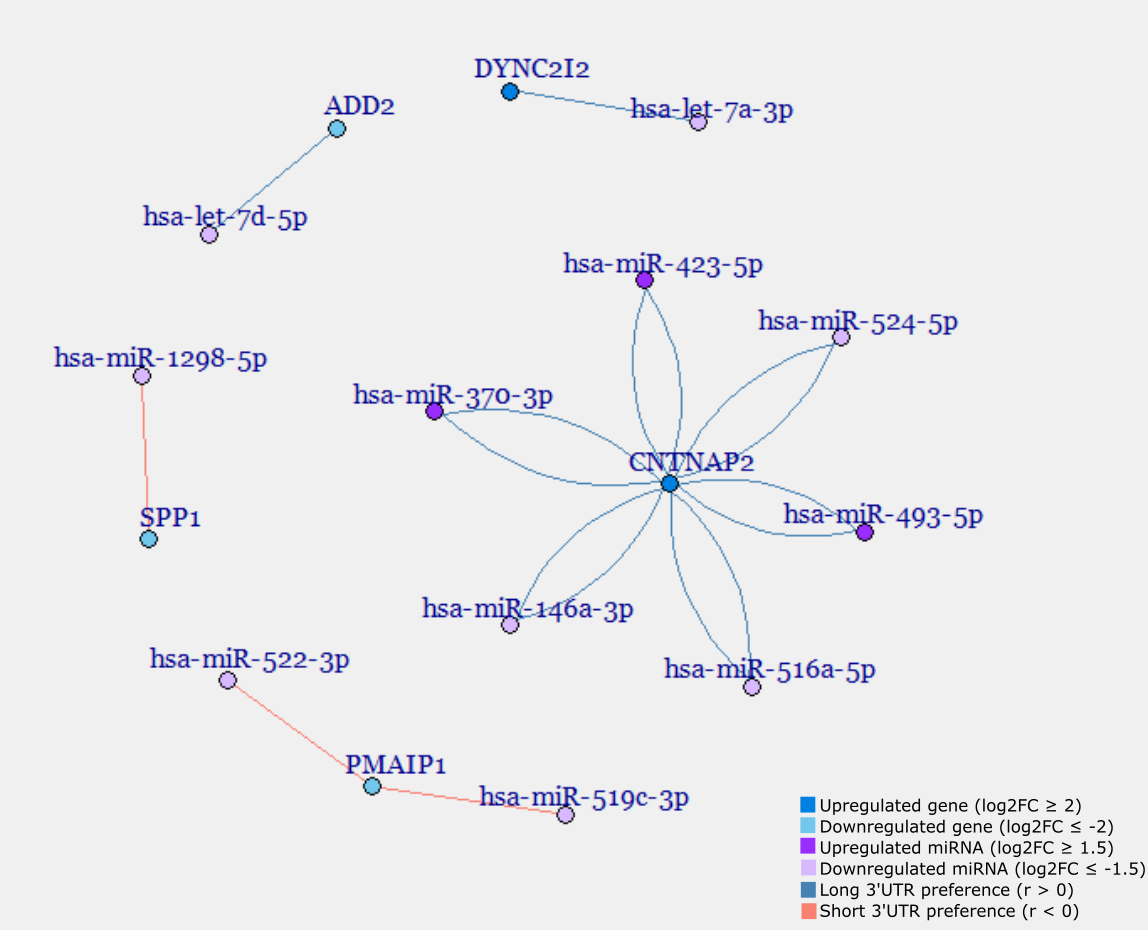

Supplement: Supplementary file 5 [file Image4.TIFF]
