# Supplementary material for: Alternative polyadenylation and dynamic 3′ UTR length is associated with polysome recruitment throughout the cardiomyogenic differentiation of hESCs
Source: Front Mol Biosci. 2024 Feb 6;11:1336336. doi: 10.3389/fmolb.2024.1336336 (PMC10877728; doi:10.3389/fmolb.2024.1336336)
Supplement: Supplementary file 3 [file Table1.DOCX]

**Supplementary Data**

Supplementary Table 1: Mean number of sequenced and mapped reads in the human genome GRCh38/hg38 v.103 in each sample, using HISAT2 as aligner.

|  | Total sequenced reads | Non mapped reads | Uniquely mapped reads | Reads mapped more than once |
| --- | --- | --- | --- | --- |
|  | 33973083.97 | 1613447.53 | 24855765.57 | 7503870.87 |
| % | 100% | 5.02% | 72.95% | 22.03% |


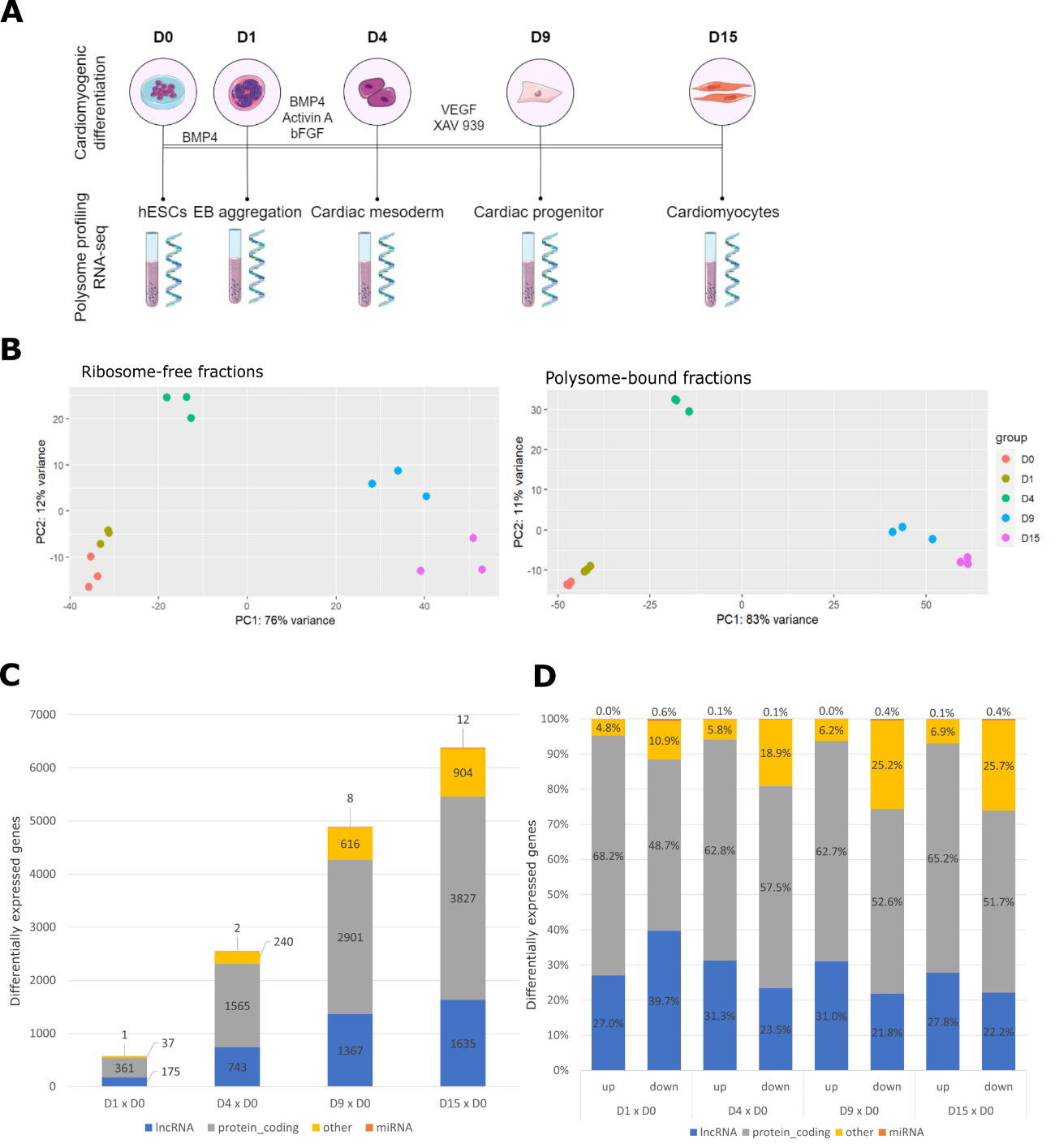


Supplementary Figure 1: Differential expression is distinct throughout the cardiomyogenic differentiation from hESCs

A: Representation of the cardiomyogenic differentiation using the embryonic body (EB) protocol, followed by polysome profiling and RNA-seq. Separation and RNA sequencing of polysome-bound and ribosome-free fractions was performed at each indicated differentiation time point. The cardiomyogenic differentiation representation was generated using Medical Art, provided by Servier, licensed under a Creative Commons Attribution 3.0 unported license.

B: Principal Components Analysis (PCA) of the polysome-bound and ribosome-free samples at each differentiation time point (n = 3). Groups are the samples of the days of cardiomyogenic differentiation (D0, D1, D4, D9 and D15).

C: Differentially expressed genes during cardiomyogenic differentiation, according to the day of differentiation, separated by gene biotypes and considering their respective 3 replicates. The four gene biotypes’ groups considered were protein coding, lncRNA, miRNA and others that don’t fall in either category. Categories provided by version v.103 of human genome GRCh38 of Ensembl.

D: Proportions of upregulated and downregulated genes separated by gene biotype during cardiomyogenic differentiation. Differential expression relative to pluripotent stage D0 as control, differential to the other time points of cardiomyogenic differentiation.


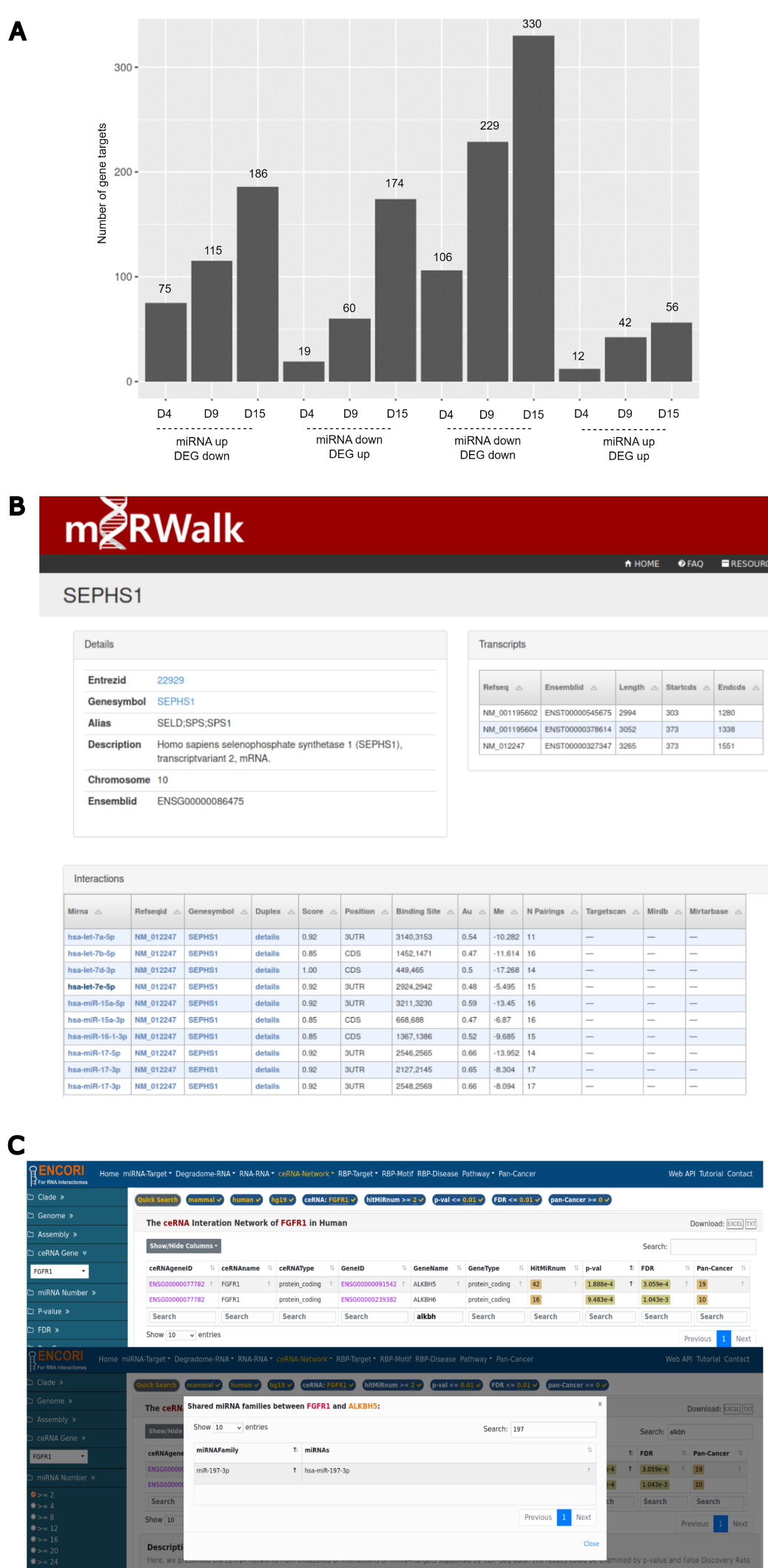


Supplementary Figure 2:

A: Modulation of differentially expressed genes targeted by differentially expressed miRNAs on their 3’ UTR region from alternative polyadenylated transcripts, all of which are present during the differentiation of hESCs to cardiomyocytes, on the days D4, D9 and D15.

B: Experimentally validated gene-miRNA target interaction from miRWalk database corroborates with our predicted results of miRNA targeting on the 3’ UTR. The miRNA hsa-let-7e-5p (in bold in the Interactions table) targets the SEPHS1 on the position of the 3’UTR.

**C:** The ceRNA interaction network of FGFR1 and ALKBH5 is connected through the miRNA family mir-197-3p, hsa-miR-197-3p. In D15, the gene FGFR1 was found downregulated with a log2FoldChange of -2.06, whilst ALKBH5 and hsa-miR-197-3p are upregulated with a log2FoldChange of 2.03 and 1.97 respectively.


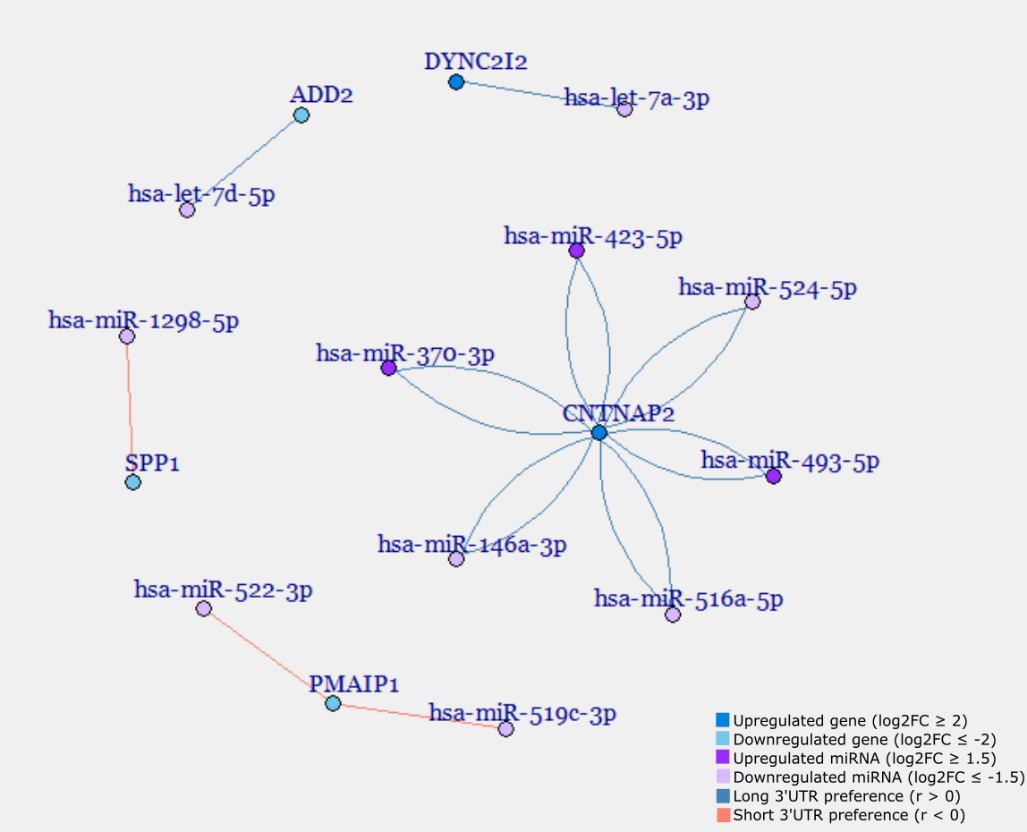


Supplementary Figure 3: Gene regulatory network of D4P of cardiomyogenic differentiation of hESC. Genes and miRNAs are vertices and transcripts are edges.

Supplementary Table 2: Summary of the gene regulatory networks constructed based on differentially expressed genes and miRNAs, and dynamic 3’UTR lengthened transcripts from time points D4, D9 and D15 of the cardiomyogenic differentiation from hESCs. Vertices indicate the gene targets and miRNAs, whereas Edges of the graphs indicate the alternative transcripts that showed dynamic 3’UTR lengthening.

|  | Vertices (genes and miRNAs) | Edges (APA isoforms) |
| --- | --- | --- |
| D4P | 16 | 17 |
| D9P | 87 | 144 |
| D15P | 146 | 210 |
